# What drives the winter behaviour and ecology of the temperate coral *Astrangia poculata?* An examination of thermal and nutritional cues

**DOI:** 10.1098/rsos.252567

**Published:** 2026-11-04

**Authors:** Taylor Lindsay, John Girard, Sean P. Grace, Randi Rotjan

**Affiliations:** ^1^ Department of Biology, Tufts University, Medford, MA, USA; ^2^ Department of Biological Sciences, University of Rhode Island, Kingston, RI, USA; ^3^ Department of Biology and Werth Center for Coastal and Marine Studies, Southern Connecticut State University, New Haven, CT, USA

**Keywords:** winter ecology, temperate coral, quiescence, *Astrangia poculata*, facultative symbiosis

## Abstract

In temperate ecosystems, accessing subtidal environments in the winter is challenging, and therefore, winter ecology and seasonal variation are understudied. For the temperate coral *Astrangia poculata*, winter is thought to be a period of biological ‘reset’, in which many cellular processes slow or stop, and the microbiome and symbiont community shift dramatically. The period of quiescence, when corals enter a dormant state with limited feeding or metabolic activity, is primarily driven by cold temperatures. However, other ecological drivers of quiescence remain unclear. Here, we documented quiescence in symbiotic and aposymbiotic colonies of *A. poculata* over a four-month winter period at two depths. First, we confirmed that quiescence is strongly associated with water temperatures under 6°C. Although we quantified a thermocline associated with depth, there was no influence of depth on quiescence. Symbiotic corals were more likely than aposymbiotic corals to enter quiescence, and higher chlorophyll *α* concentration was associated with lower quiescence, indicating a nutritional link to quiescence in *A. poculata*. Finally, we compared our results with previous quiescence observations and determined that the dormancy period is shorter in warmer winters, which has implications for the future ecology and behaviour of *A. poculata* in an era of global change.

## 1. Introduction

For sessile marine organisms such as corals, winters in temperate environments pose a significant challenge for survival. Reduced food availability, extreme low temperatures and increased storm frequency all require specialized traits and energy storage to survive. However, coastal marine ecosystems are warming in an era of global change, resulting in shorter, warmer winters [1–3]. For benthic marine invertebrates, this shift in environmental pressures may result in range expansions, altered reproductive cycles, larval dispersal and ecosystem regime shifts [3–5].

The temperate coral *Astrangia poculata* compensates for low winter temperatures by entering a period of dormancy called quiescence [6]. *Astrangia poculata* is a facultatively symbiotic coral inhabiting coastal waters from the Gulf Coast in Texas to Florida and up the Atlantic coast to Buzzards Bay, straddling Rhode Island and Massachusetts [7]. In its northerly range, it is exposed to large annual swings in water temperature (approx. 2–22°C). During quiescence, corals retract their tentacles, inflate their oral plates and become unresponsive to tactile stimuli [6]. This behaviour limits the coral's capacity for heterotrophic feeding and many cellular functions slow down or stop [8,9]. This torpor state may initiate a complete lack of nutritional input to the system for the duration of winter, depending on the symbiotic state of the coral. As a facultatively symbiotic coral, *A. poculata* individuals live within a range of symbiont densities from high (symbiotic) to low (aposymbiotic) [10]. Symbiotic individuals may still acquire photosynthetically-derived nutrition for the full year, even during dormancy, whereas aposymbiotic individuals probably do not. Quiescence is a specialized trait in *A. poculata*, as other scleractinians are unable to enter a dormant state when exposed to cold, instead suffering bleaching and mortality [11–13].

Critically, quiescence is a time of biological reset, during which the symbiont communities in *A. poculata* decrease significantly and the microbiome is significantly restructured [14–17]. Dormancy is also associated with decreased calcification and growth rates, sometimes with net tissue loss over the course of the season [6,8,9,18]. During quiescence, *A. poculata* has expanded gene families associated with feeding suppression and metabolic torpor compared to tropical corals [19]. Furthermore, gene expression changes, with enriched genes for cellular structure, protein turnover and protein homeostasis during the winter months [20,21].

Quiescence is primarily caused by low temperatures, but there is growing evidence that starvation or low food availability may also influence the onset of dormancy [22]. Laboratory experiments have documented quiescent behaviour at low temperatures; however, the exact water temperature at which quiescence occurs remains unclear. Moloney [23] documented 100% of corals entering dormancy at 5°C. In corals at 6°C, Wuitchick *et al.* [21] documented changes in behaviour. Brown *et al.* [18] found a decreasing polyp extension rate starting at 12°C and continuing to almost 0% activity at 5°C. Jacques *et al.* [9] found very low polyp activity below 6.5°C. In addition to low temperatures, quiescent behaviour has been observed under starved conditions [22,24]. Winters in temperate coastal waters are typically characterized by low primary productivity, which could lead to the simultaneous environmental pressures of cold water and low food availability. However, Narragansett Bay has a winter–spring plankton bloom which may relieve local corals from starvation pressure [25,26]. The relationship between food availability and quiescence remains unclear.

Two previous studies have documented the period of quiescence in wild populations of *A. poculata*. First, Grace [6] quantified dormancy in the intertidal and subtidal populations of Rhode Island in the winter of 2014. They found that 100% of colonies entered quiescence and that intertidal individuals entered earlier than subtidal individuals, probably owing to colder air temperature [6]. Second, Brown *et al.* [14] documented the period of quiescence while quantifying shifts in microbiome with season. Both the studies observed quiescence starting in December and continuing through the winter until spring (March–May). Both the studies also identified that dormancy occurred when temperatures were lower than 3.5–8.5°C. While these previous works have provided valuable insight into the winter ecology of *A. poculata*, there is still much to learn about this system. Most critically, both studies were limited to aposymbiotic individuals because they are more abundant. Given that quiescence limits heterotrophic feeding, symbiotically derived nutrition may play an important role in the winter ecology of *A. poculata,* especially given recent evidence that photosynthesis continues during quiescence [23]. Changes in light availability with depth may further affect the coral's capacity for photosynthesis during dormancy.

To identify the effects of symbiont state, water quality and water temperature on quiescence in *A. poculata*, we surveyed the Rhode Island population in the winter of 2025. First, we investigated the effect of depth on quiescence in *A. poculata,* hypothesizing no effect because frequent winter storms mix the water column, creating temperature stability. Second, we examined the relationship between symbiosis and dormancy. Because of the significant decrease in symbiont populations in symbiotic individuals over the winter [17], we predicted that symbiotic colonies would experience a greater nutritional loss with changing seasons. Therefore, symbiotic colonies may enter quiescence earlier and for a longer period than aposymbiotic individuals. Third, we explored possible water quality triggers of quiescence, hypothesizing that both water temperature and chlorophyll *α* concentration would have negative relationships with the onset and duration of dormancy. While low water temperature has previously been tied to quiescence, we hypothesized that low food availability in winter may also affect behaviour and use surface chlorophyll *α* concentration as a proxy for food availability. Finally, we used historical quiescence data to determine whether warmer, shorter winters affected the quiescence period of *A. poculata*.

## 2. Methods

### 2.1. Quiescence monitoring

Quiescence was monitored bi-weekly for four months in the winter of 2025 at Fort Wetherill State Park in Rhode Island, USA (41.477764° N, 71.359622° W), at the northern range of the species (supplementary material, figure S1). To test the effect of depth on quiescence, divers returned to the same subtidal locations at 6 and 11.5 m on each sampling date. At each depth, quiescence status was quantified in 10 colonies of aposymbiotic and 10 colonies of symbiotic corals (*n* = 40 per sampling date) to determine the effect of symbiotic morphotype. Individual coral colonies were randomly selected on each date. Because the same locations were used each time, it is likely that some of the same individual corals were measured on multiple occasions. *In situ* colony colour has previously been determined as a good indicator of symbiont density in *A. poculata* in the summer; however, the decrease in symbiont density in symbiotic colonies during the winter may have affected identification [8,17,27].

Quiescence was quantified on a scale from 0 to 3 according to the descriptions in table 1 using both visual assessment and tactile response. Divers first conducted a visual assessment of the corals to identify the degree of tentacle extension at rest. Then, a tactile response was initiated by probing each colony a maximum of two times.

**Table 1.** Quiescence score guide is based on visual assessment and tactile response.

score	visual assessment	tactile response
0	tentacles extended	tentacles react when touched
1	tentacles extended	tentacles do not react when touched
2	tentacles partially retracted	tentacles do not react when touched
3	tentacles fully retracted, puffed and inflated oral disc	tentacles do not react when touched

### 2.2. Temperature, chlorophyll and historical data

Water temperature data were recorded using loggers deployed at depth and retrieved from nearby surface buoy data. Onset HOBO loggers (MX2202) were deployed at each focal depth (6 and 11.5 m) for the duration of the monitoring period. These data were coupled with sea surface temperature extracted from National Oceanic and Atmospheric Administration (NOAA) buoys (stations NWPR1 and BZBM3; supplementary material, figure S2). To capture the water conditions prior to sampling, the temperature used for each quiescence sampling day was calculated by averaging values from one week preceding the sampling date for each depth, using data from the deployed Onset HOBO loggers.

Sea surface chlorophyll data were downloaded from the Suomi National Polar-orbiting Partnership's Visible Infrared Imaging Radiometer Suite and Ocean and Land Color Instrument via NOAA's Environmental Research Divisions Data Access Program [28,29]. Chlorophyll *α* concentration (mg m^–3^) was extracted from the mouth of Narragansett Bay (approx. 41.4° to 41.5° N and 71.45° to 71.35° W; supplementary material, figure S1). Daily means were calculated by averaging all pixels and the mean chlorophyll *α* concentration for each quiescence sampling day was calculated by averaging values from one week preceding the sampling date.

To compare the period of quiescence with previous years, initial and final quiescence dates were extracted from the 2024–2025 dataset and paired with data from the winters of 2013–2014 [6] and 2020–2021 [14]. The corals from Brown *et al.* [14] were observed at Woods Hole, MA whereas all other observations occurred in Narragansett Bay, RI (supplementary material, figure S1). For this comparison, sea surface temperature extracted from local buoys was used to maintain sampling consistency.

### 2.3. Statistical analysis

All statistical analyses were conducted using R version 2025.05.1. Student's *t*-tests were used to compare the four-month temperature data between the surface, shallow and deep environments.

Because quiescence scores are based on ordered categorical rankings, we used an ordinal statistical approach to build a model exploring the impacts of depth, water temperature and morphotype on *A. poculata* quiescence. Proportional odds logistic regression was implemented using the *polr* function from the MASS package (version 7.3.65) on the following model: quiescence score ∼ depth + morphotype + water temperature + chlorophyll *α* [30]. For this model, both chlorophyll *α* and temperature were the mean of all values for one week preceding the sampling date. Chlorophyll *α* values were the same regardless of depth because only surface observations are available, but temperature was taken from the HOBO loggers *in situ* at each sampling depth.

## 3. Results

Over the four-month monitoring period, the surface water (0 m) was significantly colder than the deep site (11.5 m, *p* = 0.036; figure 1A). However, the shallow (6 m) observation site was not significantly different from the surface (0 m, *p* = 0.08) or deep (11.5 m, *p* = 0.65) sites. There was a temperature inversion over the course of the study period, with colder surface water than subtidal sites from January to mid-March, followed by a switch to warmer surface water in the spring (figure 1B). Surface and subtidal water temperatures were below the 6°C threshold for quiescence from mid-January to mid-March. Chlorophyll *α* concentration experienced blooms in January and April, with a slightly depressed period during the coldest months (figure 1C).

**Figure 1.**
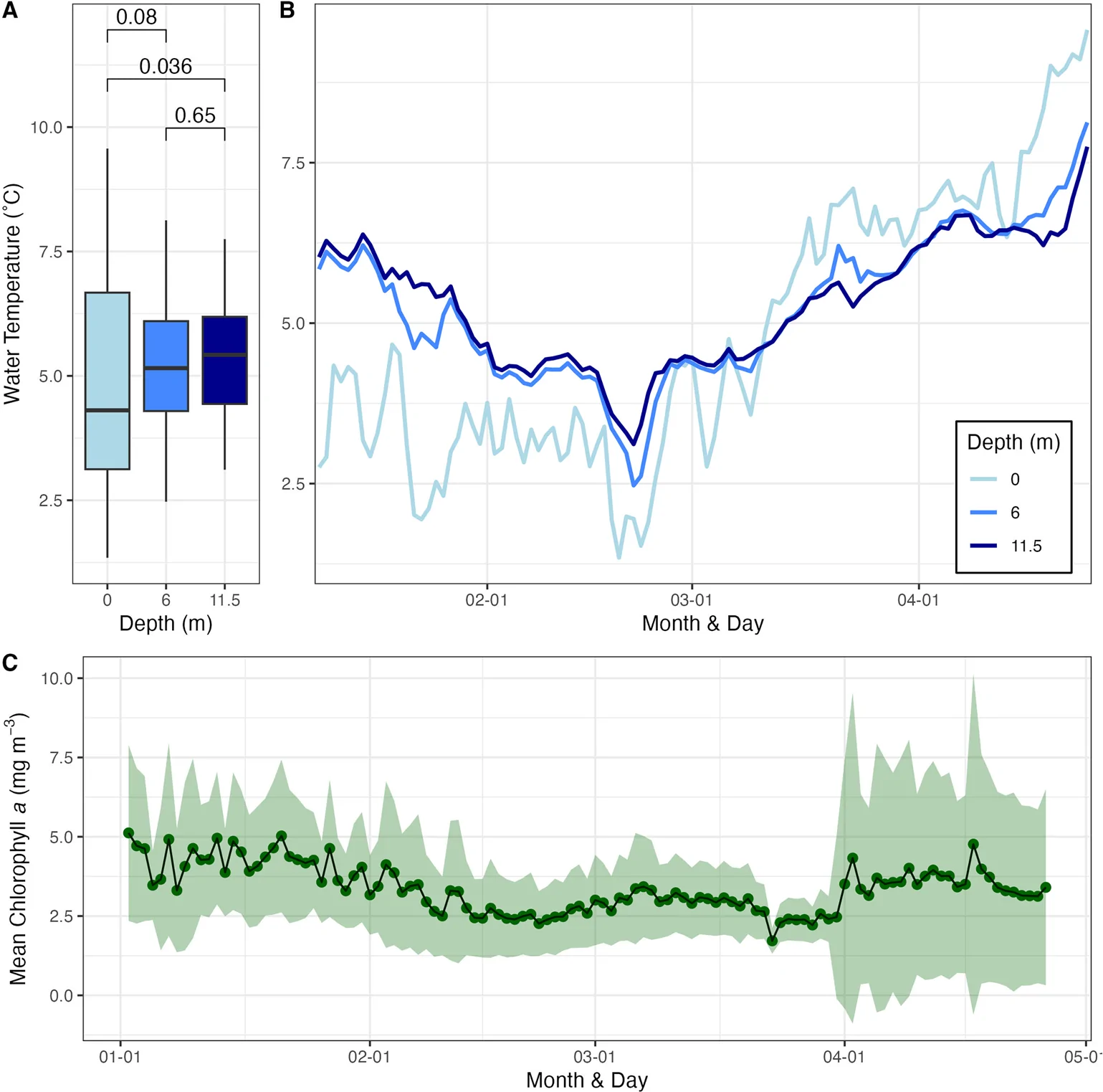
Water temperature in Narragansett Bay, RI during the winter of 2025 in surface (NOAA Buoy Stations NWPR1), shallow (6 m, Onset HOBO) and deep (11.5 m, Onset HOBO) water. (A) Mean daily water temperature from January through to May was significantly colder on the surface than in the shallow or deep sites. *p*-values from Student's *t*-tests are presented for each pair of depths. (B) Mean daily water temperature over the duration of the study revealed a temperature inversion, with surface water colder than bottom water from January to March, followed by a switch to warmer surface waters in early March. (C) Mean daily sea surface chlorophyll *α* concentration in Narragansett Bay.

Across all three years in which *A. poculata* quiescence has been documented, the surface water temperature reached weekly means of 6°C or less before or at the time when quiescence was first observed (figure 2). However, the period of cold water (less than 6°C) has shortened over time with each documented observation of *A. poculata* quiescence. In 2014, corals exhibited quiescence for over 23 weeks, followed by almost 15 weeks of dormancy in 2021 and just over six weeks in 2025 [6,14]. This suggests that the shorter cold period has resulted in shorter quiescence periods in corals in Rhode Island and Massachusetts.

**Figure 2.**
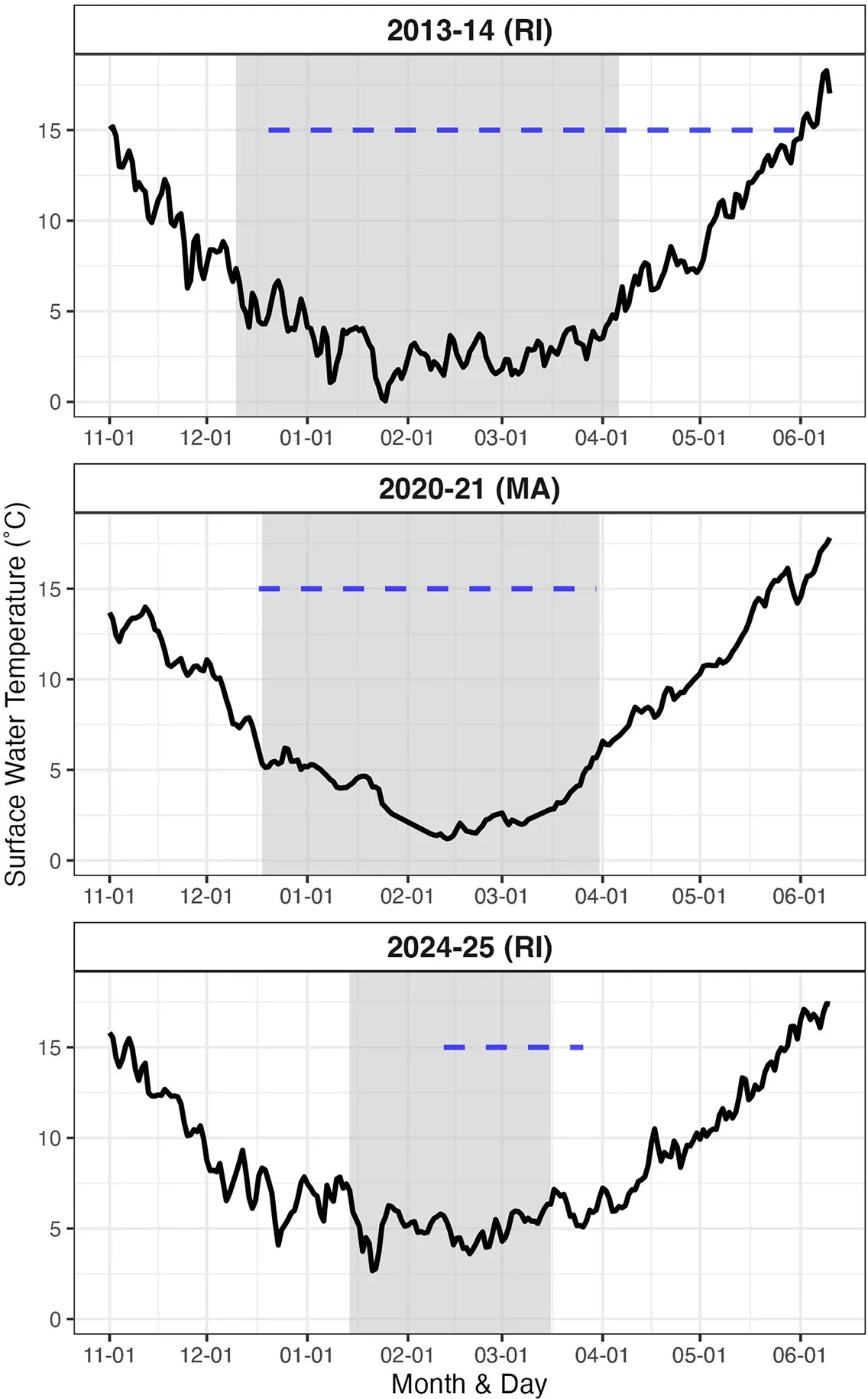
Mean daily surface water temperature (solid black lines) from the three years of interest in either Rhode Island or Massachusetts. Grey shading represents the period during which the mean weekly surface temperature was less than 6°C. Blue dotted lines represent the duration of observed quiescence. Despite the cold water, few colonies of *A. poculata* entered quiescence in the winter of 2025 (figure 3A). Corals were observed with a score of one or greater for a period of six weeks (12 February 2025 to 26 March 2025), demonstrating reduced polyp activity during this time. The maximum percentage of corals exhibiting signs of quiescence never exceeded 37.5% (15 out of 40 colonies).

Proportional odds logistic regression revealed that morphotype, water temperature and chlorophyll, but not depth, significantly impacted quiescence in *A. poculata*. Morphotype had a significant impact on dormancy (*p* = 0.041; figure 3C; table 2) with symbiotic individuals 187% more likely to have a higher quiescence score than aposymbiotic individuals. Between the shallow and deep sites observed in the winter of 2025, depth did not significantly affect quiescence in *A. poculata* (*p* = 0.247; figure 3B; table 2). Lower water temperature significantly increased the odds of dormancy (*p* = 0.0007; table 2). For every 1°C decrease in water temperature, the odds of being in a higher quiescence category increased by 262%. Finally, sea surface chlorophyll had a significant negative relationship with dormancy (*p* = 0.018; table 2). For every 1 mg m^–3^ increase in chlorophyll *α* concentration, the odds of quiescence decreased by 83%.

**Figure 3.**
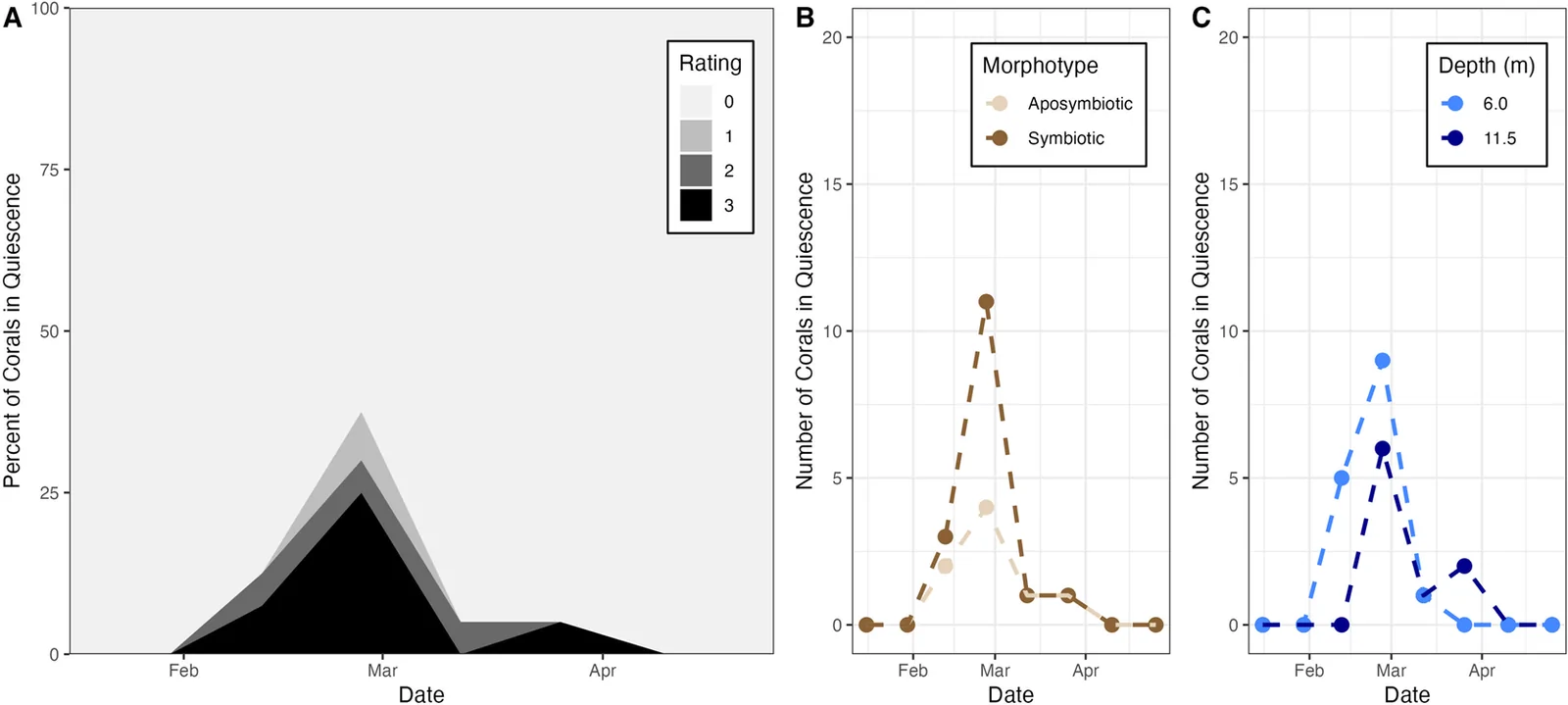
Quiescence in *A. poculata* over the winter of 2025. (A) Percentage of corals in dormancy was relatively low for most of the observation period, with a peak of 37.5% in late February. (B) A greater number of symbiotic corals entered quiescence in late February than aposymbiotic corals. This pattern held at both depths (supplementary material, figure S3). (C) Corals in the shallow habitat (6 m) entered quiescence slightly earlier and in slightly higher abundance than those in the deep habitat (11.5 m), although this relationship was not significant. Both panels (B) and (C) count corals in stages 1–3 as in quiescence.

**Table 2.** Results of a proportional odds logistic regression using the following model: quiescence ranking ∼ depth + morphotype + water temperature + chlorophyll. Significant values are in bold and indicated by an asterisk.

predictor	value (log-odds)	s.e.	odds ratio	*t*-value	*p*-value
depth	−0.03	0.02	0.97	−1.16	0.247
ecotype	1.06	0.52	2.87	2.04	0.041*
mean water temp	−1.29	0.38	0.28	−3.39	0.0007*
mean chlorophyll	−1.82	0.77	0.16	−2.36	0.018*

## 4. Discussion

### 4.1. Quiescence is shortening under warming ocean temperatures

Over the past 11 years, the quiescence period of *A. poculata* has shortened, most probably in response to warming sea surface temperature (supplementary material, figure S2). The 23 week period of dormancy in 2014 was dramatically reduced to just over six weeks in the winter of 2025. Furthermore, 100% of the 2014 individuals entered quiescence, whereas fewer than 50% of the corals exhibited dormant behaviour at any given time in 2025, suggesting that some individuals never entered quiescence [6]. There was a four-week lag between cold water below 6°C and the first observations of quiescence in 2025. By contrast, dormancy was first observed within 10 days of 6°C water in both 2014 and 2021. Although the period of less than 6°C water has decreased since 2014, this does not explain the delay in quiescence observed in 2025. Anecdotally, the authors have observed little to no quiescence in the warm winters prior to the winter of 2025 (2023 and 2024). It is possible that repeated warm winters in which conditions did not prompt dormancy have decreased the likelihood that corals will enter quiescence, even when the temperature meets the expected threshold.

Under continued ocean warming conditions, the period and likelihood of quiescence are expected to decrease, causing biological costs and benefits to the coral. Higher water temperature has been linked to faster growth rates and greater calcification in *A. poculata* [8,9,31]. Thus, warmer waters could lead to larger, faster-growing colonies, especially if growth continues over the winter season. However, if corals do not enter quiescence, they must maintain their metabolic activity, which has associated energetic costs. Without sufficient energy resources available owing to lower light levels and less food in the winter, the corals may not be able to sustain their metabolic needs, resulting in mortality or tissue loss. Warming waters will also probably result in a northward range expansion in *A. poculata* [32]. Until now, *A. poculata* has not been observed living north of Cape Cod in the Gulf of Maine. However, as climate change continues to warm coastal waters and seasonal pressures become less severe, a northward range expansion is likely [32]. Finally, in scleractinian corals, temperature is a strong modulator of gametogenesis and spawning [33,34]. According to Szmant-Froelich *et al.* [35], gametogenesis in *A. poculata* is closely tied to seasonal water temperatures. Males typically have close to 0% spermaries through the winter and females experience a gradual decrease in oocytes during the coldest months (December through to March). As the water temperatures begin to increase in the spring, both spermatocyte and oocyte production increase. Without the pressure of low-temperature or metabolic shutdown of quiescence, *A. poculata* may have more energy to invest in reproduction, leading to earlier gametogenesis or a larger energetic investment.

### 4.2. Depth and light do not affect quiescence

Quiescence did not differ between depths in the winter of 2025. Overall, the water column was relatively well mixed and the water temperature between 6 and 11.5 m was not significantly different. This suggests that other water quality metrics, such as food availability, pollutants, dissolved nutrients and salinity, were also well mixed and did not differ greatly between the observation sites. Thus, the main environmental variable that differentiated shallow and deep corals was light availability. Previous work at this site has illustrated light gradients during the summer [36]. We anticipate that lower plankton abundance in the winter leads to lower water turbidity and, therefore, less dramatic attenuation along the depth gradient. Thus, the environmental factors that differ along depth gradients are probably not distinct enough in winter to evidence a change in coral quiescence across depths and scale observed.

### 4.3. Morphotype and nutritional pressures

Symbiotic colonies of *A. poculata* are more likely to enter quiescence than aposymbiotic individuals. In winter, symbiotic colonies experience a decrease in their symbiont load, essentially to the densities observed in the aposymbiotic colonies [17]. Meanwhile, aposymbiotic colonies do not experience the same change. We hypothesized that this shift in trophic dynamics may drive the relationship between morphotype and quiescence. Although symbionts do not contribute significantly to the energy acquisition of *A. poculata* [27], they provide a variety of benefits, including greater survival and growth, improved wound healing and nutrient assimilation [8,10,24,32,37–39]. Without the benefits provided by symbionts, corals may enter quiescence earlier to prevent starvation or tissue loss.

Another possible driver of quiescence in *A. poculata* is limited food availability in the winter, as previously suggested by Grace [6]. During periods of low temperature and low light, primary production in coastal waters decreases, resulting in lower densities of planktonic food for temperate corals [25]. Under laboratory starvation conditions, *A. poculata* has a significant decrease in polyp activity, photosynthetic efficiency and respiration [24,37,40,41]. These traits suggest a quiescence response; however, the interaction between cold, starvation and quiescence has not yet been explored. Narragansett Bay is characterized by a winter–spring plankton bloom, which may sustain some food availability throughout the winter, compared to other open-ocean sites [25,26]. Despite this, we found that sea surface chlorophyll *α* concentration was negatively associated with quiescence, suggesting that as chlorophyll *α* increased, food availability may have been greater and corals were less likely to enter dormancy. This finding supports the hypothesis that food availability contributes to nutritional quiescence in *A. poculata*; however, much more work is required to understand the nutritional effects on quiescence, as chlorophyll *α* is only a proxy for actual food availability.

Other cnidarians may experience nutritional quiescence when food availability is low, especially in the Mediterranean, where summer is characterized by low food availability. Most Mediterranean benthic suspension feeders, including over 100 species of cnidarians, reproduce, grow and feed primarily in winter when food is abundant [42]. In summer, activity decreases and some species enter dormancy. For example, in the octocoral *Alcyonium acaule*, up to 90% of colonies entered a contracted, dormant state in summer [43]. However, most corals inhabit oligotrophic waters where food availability is low year-round. These tropical corals rely heavily on their symbionts for nutrition, thus avoiding the effects of low food availability that may lead to nutritional quiescence.

### 4.4. Future directions for winter ecology

Despite its growing popularity as an experimental system, the ecology of *A. poculata* remains poorly understood [7]. In particular, the role of seasonal swings in temperature, light and food availability, macroalgae and other water quality and ecology metrics remain largely unexplored. While the onset of quiescence is related to water temperature, the exact temperature at which this behaviour occurs remains unclear—probably because temperature alone does not dictate the onset of quiescence. Combined with our findings linking morphotype and chlorophyll with dormancy, the growing evidence for nutritional quiescence raises important questions about the metabolic role of dormancy in *A. poculata* and the adaptive benefits of this trait.

To better understand the effects of winter on the biology of *A. poculata*, we can turn to its southern populations for comparison. How does reproduction, growth and physiology of this species differ in populations inhabiting warmer waters without extreme low winter temperatures? Quiescence in *A. poculata* is a unique trait that allows it to survive extreme seasonal variation and is found in very few cnidarian species. As climate change rapidly alters the environmental conditions and seasonality of coastal systems, it is more important than ever to develop baselines and understand the impacts of winter ecology on the biology of marine invertebrates.

## Supplementary Material



## Data Availability

All raw data and code are available on GitHub [44] and have been archived on Zenodo [45]. Supplementary material is available online [46].
