# Supplementary material for: What drives the winter behaviour and ecology of the temperate coral Astrangia poculata? An examination of thermal and nutritional cues
Source: R Soc Open Sci. 2026 Nov 4;13(11):252567. doi: 10.1098/rsos.252567 (PMC13730659; doi:10.1098/rsos.252567)
Supplement: Supplementary file 1 [file rsos.252567.zip › rsos.252567/rsos252567_si_001.docx]

**SUPPLEMENTAL MATERIALS FOR:**

What drives the winter behavior and ecology of the temperate coral *Astrangia poculata?* An examination of thermal and nutritional cues

Taylor R. Lindsay, John Girard, Sean Grace, Randi D. Rotjan

Royal Society Open Science, 10.1098/rsos.252567

**Figure S1.** Map of the study area in southern Rhode Island and Massachusetts. The red dot represents the study location for the 2024-25 and 2013-14 surveys (Fort Wetherill State Park, Station NWPR1). The orange triangle represents the 2020-21 survey (Marine Biological Laboratory, Station BZBM3). The yellow square represents the area from which sea surface chlorophyll was calculated.

**Figure S2.** Mean daily water temp from NOAA buoys in Rhode Island (Station NWPR1) and Massachusetts (Station BZBM3) for 20 years (black lines). A linear regression (blue line) shows that temperature is significantly increasing at both sites over time (p <0.001).

**Figure S3.** Quiescence over the winter of 2025 between morphotypes, separated by site depth. Corals in stages 1-3 were counted as in quiescence.
